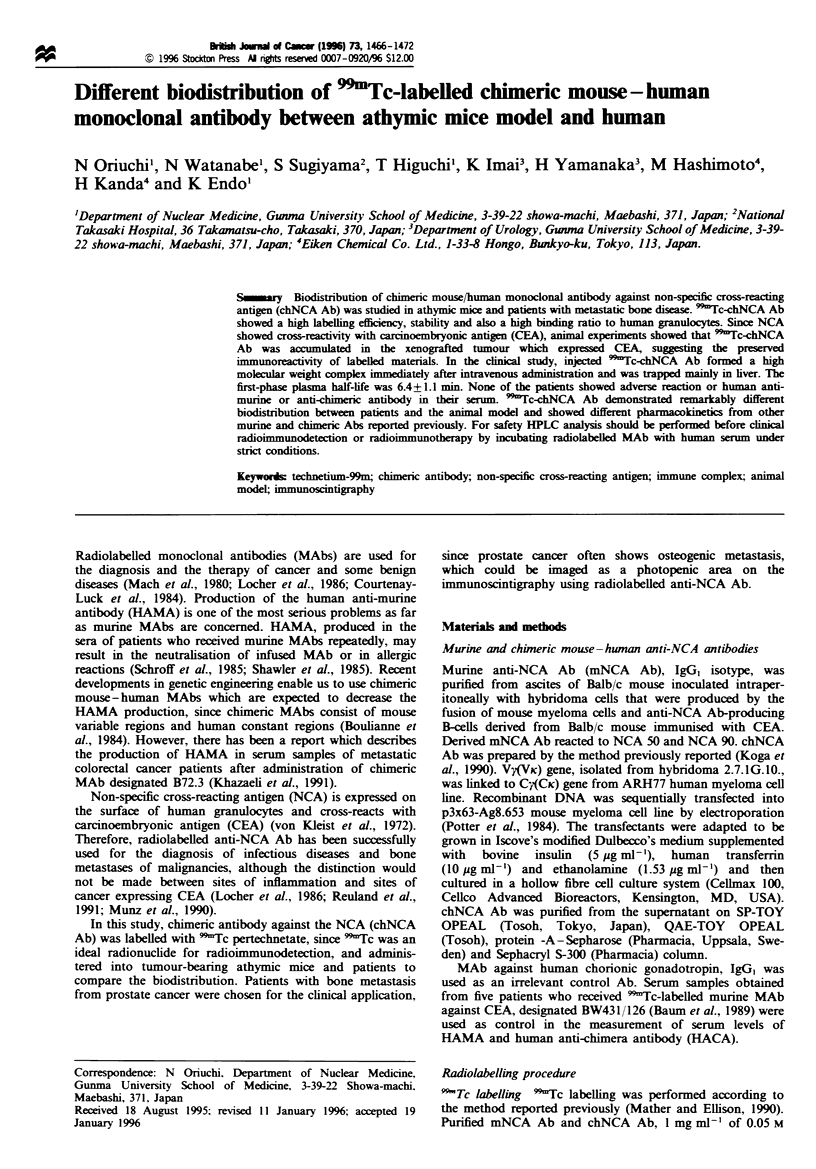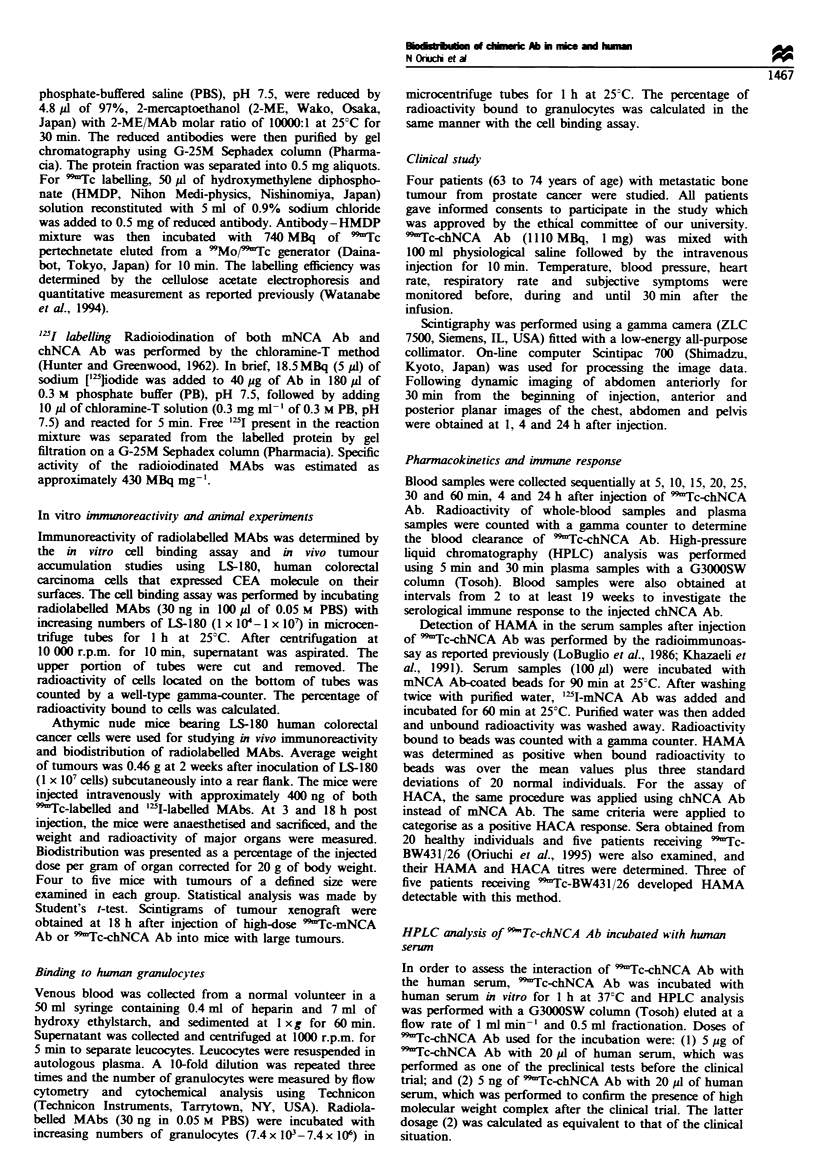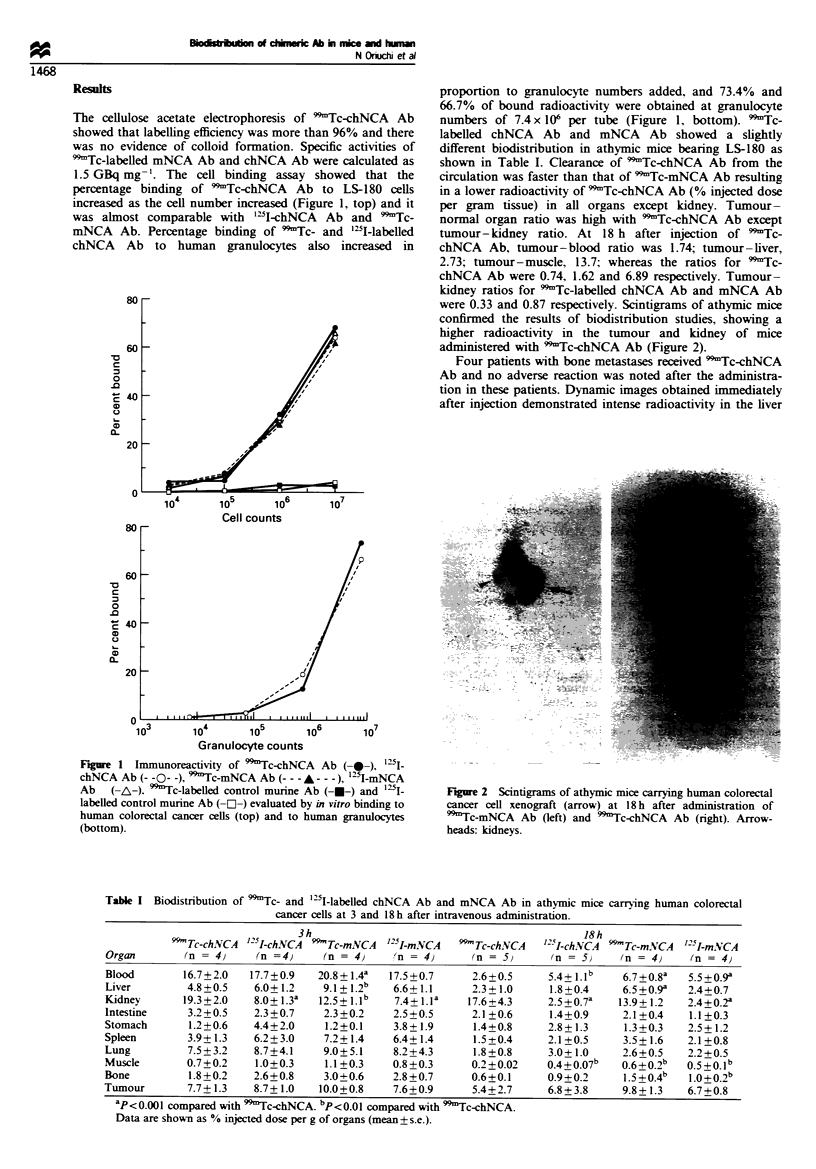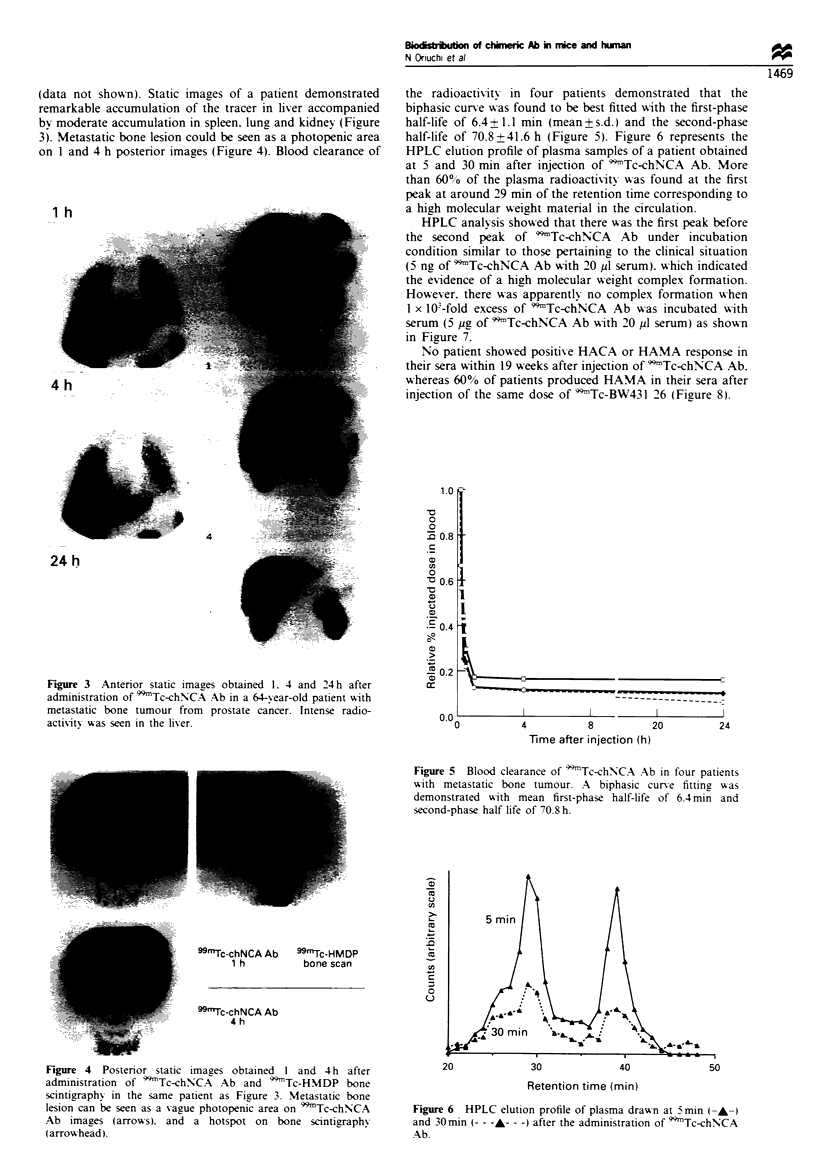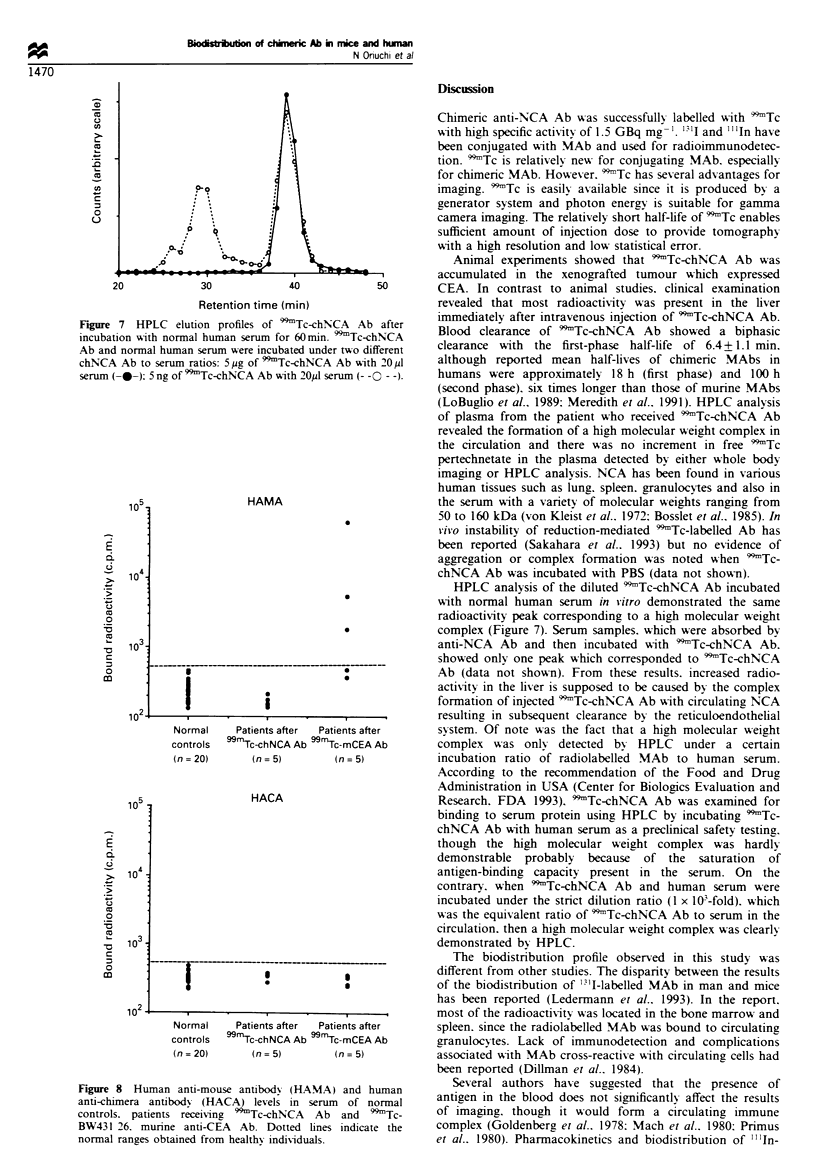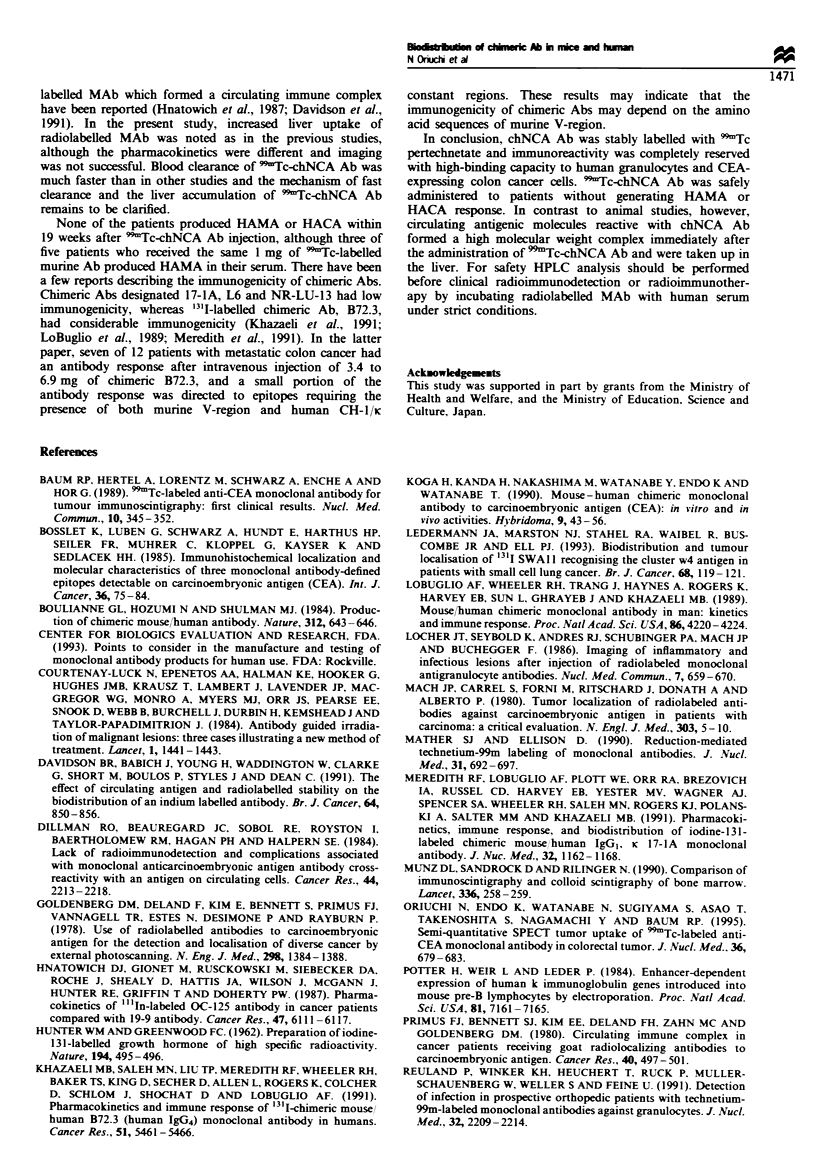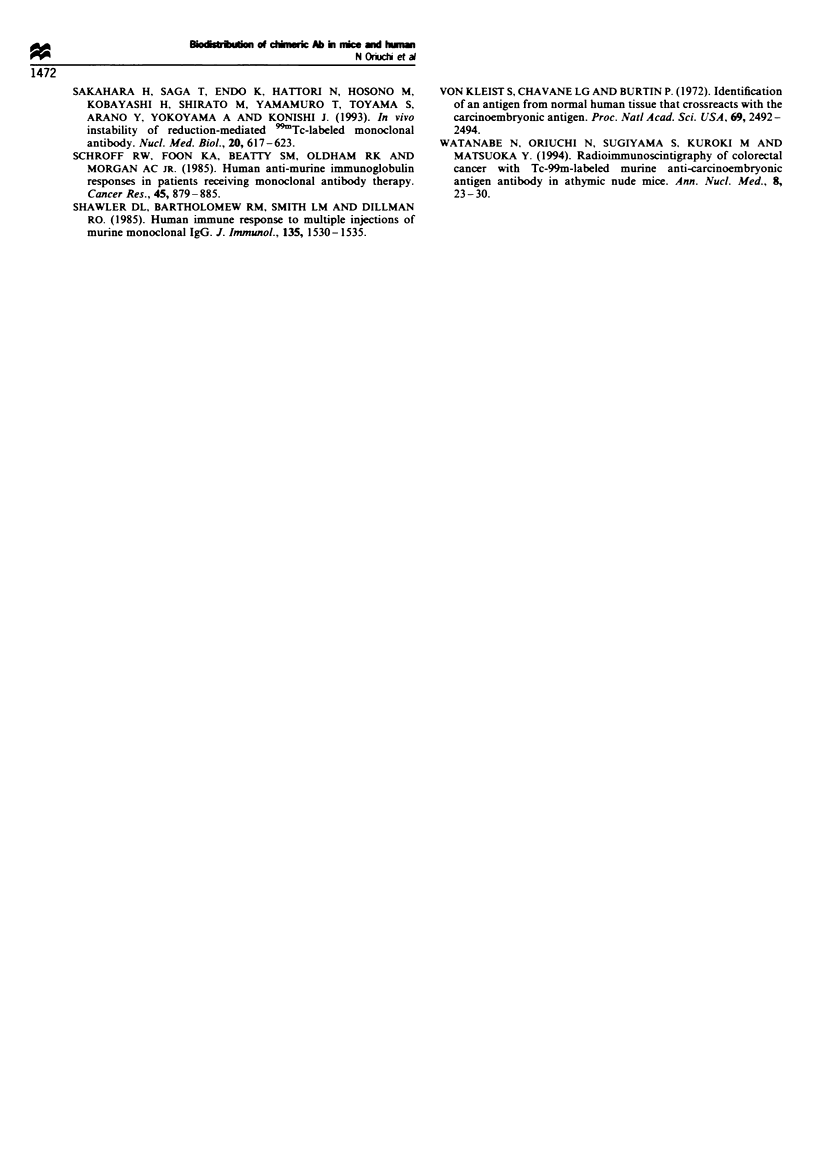# Different biodistribution of 99mTc-labelled chimeric mouse-human monoclonal antibody between athymic mice model and human.

**DOI:** 10.1038/bjc.1996.278

**Published:** 1996-06

**Authors:** N. Oriuchi, N. Watanabe, S. Sugiyama, T. Higuchi, K. Imai, H. Yamanaka, M. Hashimoto, H. Kanda, K. Endo

**Affiliations:** Department of Nuclear Medicine, Gunma University School of Medicine, Maebashi, Japan.

## Abstract

**Images:**


British Journal of Cancer (1996) 73, 1466-1472

© 1996 Stockton Press All rights reserved 0007-0920/96 $12.00

### Different biodistribution of $^{99m}$Tc-labelled chimeric mouse-human monoclonal antibody between athymic mice model and human

N Oriuchi[1], N Watanabe[1], S Sugiyama[2], T Higuchi[1], K Imai[3], H Yamanaka[3], M Hashimoto[4], H Kanda[4] and K Endo[1]

[1]*Department of Nuclear Medicine, Gunma University School of Medicine, 3-39-22 showa-machi, Maebashi, 371, Japan;* [2]*National Takasaki Hospital, 36 Takamatsu-cho, Takasaki, 370, Japan;* [3]*Department of Urology, Gunma University School of Medicine, 3-39-22 showa-machi, Maebashi, 371, Japan;* [4]*Eiken Chemical Co. Ltd., 1-33-8 Hongo, Bunkyo-ku, Tokyo, 113, Japan.*

**Summary** Biodistribution of chimeric mouse/human monoclonal antibody against non-specific cross-reacting antigen (chNCA Ab) was studied in athymic mice and patients with metastatic bone disease. $^{99m}$Tc-chNCA Ab showed a high labelling efficiency, stability and also a high binding ratio to human granulocytes. Since NCA showed cross-reactivity with carcinoembryonic antigen (CEA), animal experiments showed that $^{99m}$Tc-chNCA Ab was accumulated in the xenografted tumour which expressed CEA, suggesting the preserved immunoreactivity of labelled materials. In the clinical study, injected $^{99m}$Tc-chNCA Ab formed a high molecular weight complex immediately after intravenous administration and was trapped mainly in liver. The first-phase plasma half-life was 6.4±1.1 min. None of the patients showed adverse reaction or human anti-murine or anti-chimeric antibody in their serum. $^{99m}$Tc-chNCA Ab demonstrated remarkably different biodistribution between patients and the animal model and showed different pharmacokinetics from other murine and chimeric Abs reported previously. For safety HPLC analysis should be performed before clinical radioimmunodetection or radioimmunotherapy by incubating radiolabelled MAb with human serum under strict conditions.

**Keywords:** technetium-99m; chimeric antibody; non-specific cross-reacting antigen; immune complex; animal model; immunoscintigraphy

Radiolabelled monoclonal antibodies (MAbs) are used for the diagnosis and the therapy of cancer and some benign diseases (Mach *et al.*, 1980; Locher *et al.*, 1986; Courtenay-Luck *et al.*, 1984). Production of the human anti-murine antibody (HAMA) is one of the most serious problems as far as murine MAbs are concerned. HAMA, produced in the sera of patients who received murine MAbs repeatedly, may result in the neutralisation of infused MAb or in allergic reactions (Schroff *et al.*, 1985; Shawler *et al.*, 1985). Recent developments in genetic engineering enable us to use chimeric mouse-human MAbs which are expected to decrease the HAMA production, since chimeric MAbs consist of mouse variable regions and human constant regions (Boulianne *et al.*, 1984). However, there has been a report which describes the production of HAMA in serum samples of metastatic colorectal cancer patients after administration of chimeric MAb designated B72.3 (Khazaeli *et al.*, 1991).

Non-specific cross-reacting antigen (NCA) is expressed on the surface of human granulocytes and cross-reacts with carcinoembryonic antigen (CEA) (von Kleist *et al.*, 1972). Therefore, radiolabelled anti-NCA Ab has been successfully used for the diagnosis of infectious diseases and bone metastases of malignancies, although the distinction would not be made between sites of inflammation and sites of cancer expressing CEA (Locher *et al.*, 1986; Reuland *et al.*, 1991; Munz *et al.*, 1990).

In this study, chimeric antibody against the NCA (chNCA Ab) was labelled with $^{99m}$Tc pertechnetate, since $^{99m}$Tc was an ideal radionuclide for radioimmunodetection, and administered into tumour-bearing athymic mice and patients to compare the biodistribution. Patients with bone metastasis from prostate cancer were chosen for the clinical application, since prostate cancer often shows osteogenic metastasis, which could be imaged as a photopenic area on the immunoscintigraphy using radiolabelled anti-NCA Ab.

Correspondence: N Oriuchi, Department of Nuclear Medicine, Gunma University School of Medicine, 3-39-22 Showa-machi, Maebashi, 371, Japan
Received 18 August 1995; revised 11 January 1996; accepted 19 January 1996

### Materials and methods

### *Murine and chimeric mouse-human anti-NCA antibodies*

Murine anti-NCA Ab (mNCA Ab), $IgG_1$ isotype, was purified from ascites of Balb/c mouse inoculated intraperitoneally with hybridoma cells that were produced by the fusion of mouse myeloma cells and anti-NCA Ab-producing B-cells derived from Balb/c mouse immunised with CEA. Derived mNCA Ab reacted to NCA 50 and NCA 90. chNCA Ab was prepared by the method previously reported (Koga *et al.*, 1990). $V\gamma(V\kappa)$ gene, isolated from hybridoma 2.7.1G.10., was linked to $C\gamma(C\kappa)$ gene from ARH77 human myeloma cell line. Recombinant DNA was sequentially transfected into p3x63-Ag8.653 mouse myeloma cell line by electroporation (Potter *et al.*, 1984). The transfectants were adapted to be grown in Iscove's modified Dulbecco's medium supplemented with bovine insulin (5 $\mu$g ml$^{-1}$), human transferrin (10 $\mu$g ml$^{-1}$) and ethanolamine (1.53 $\mu$g ml$^{-1}$) and then cultured in a hollow fibre cell culture system (Cellmax 100, Cellco Advanced Bioreactors, Kensington, MD, USA). chNCA Ab was purified from the supernatant on SP-TOY OPEAL (Tosoh, Tokyo, Japan), QAE-TOY OPEAL (Tosoh), protein -A-Sepharose (Pharmacia, Uppsala, Sweden) and Sephacryl S-300 (Pharmacia) column.

MAb against human chorionic gonadotropin, $IgG_1$ was used as an irrelevant control Ab. Serum samples obtained from five patients who received $^{99m}$Tc-labelled murine MAb against CEA, designated BW431/126 (Baum *et al.*, 1989) were used as control in the measurement of serum levels of HAMA and human anti-chimera antibody (HACA).

### *Radiolabelling procedure*

*$^{99m}$Tc labelling* $^{99m}$Tc labelling was performed according to the method reported previously (Mather and Ellison, 1990). Purified mNCA Ab and chNCA Ab, 1 mg ml$^{-1}$ of 0.05 M

phosphate-buffered saline (PBS), pH 7.5, were reduced by 4.8 μl of 97%, 2-mercaptoethanol (2-ME, Wako, Osaka, Japan) with 2-ME/MAb molar ratio of 10000:1 at 25°C for 30 min. The reduced antibodies were then purified by gel chromatography using G-25M Sephadex column (Pharmacia). The protein fraction was separated into 0.5 mg aliquots. For $^{99m}$Tc labelling, 50 μl of hydroxymethylene diphosphonate (HMDP, Nihon Medi-physics, Nishinomiya, Japan) solution reconstituted with 5 ml of 0.9% sodium chloride was added to 0.5 mg of reduced antibody. Antibody-HMDP mixture was then incubated with 740 MBq of $^{99m}$Tc pertechnetate eluted from a $^{99}$Mo/$^{99m}$Tc generator (Dainabot, Tokyo, Japan) for 10 min. The labelling efficiency was determined by the cellulose acetate electrophoresis and quantitative measurement as reported previously (Watanabe *et al.*, 1994).

*$^{125}$I labelling* Radioiodination of both mNCA Ab and chNCA Ab was performed by the chloramine-T method (Hunter and Greenwood, 1962). In brief, 18.5 MBq (5 μl) of sodium [$^{125}$I]iodide was added to 40 μg of Ab in 180 μl of 0.3 M phosphate buffer (PB), pH 7.5, followed by adding 10 μl of chloramine-T solution (0.3 mg ml$^{-1}$ of 0.3 M PB, pH 7.5) and reacted for 5 min. Free $^{125}$I present in the reaction mixture was separated from the labelled protein by gel filtration on a G-25M Sephadex column (Pharmacia). Specific activity of the radioiodinated MAbs was estimated as approximately 430 MBq mg$^{-1}$.

### In vitro *immunoreactivity and animal experiments*

Immunoreactivity of radiolabelled MAbs was determined by the *in vitro* cell binding assay and *in vivo* tumour accumulation studies using LS-180, human colorectal carcinoma cells that expressed CEA molecule on their surfaces. The cell binding assay was performed by incubating radiolabelled MAbs (30 ng in 100 μl of 0.05 M PBS) with increasing numbers of LS-180 ($1 \times 10^4 - 1 \times 10^7$) in microcentrifuge tubes for 1 h at 25°C. After centrifugation at 10 000 r.p.m. for 10 min, supernatant was aspirated. The upper portion of tubes were cut and removed. The radioactivity of cells located on the bottom of tubes was counted by a well-type gamma-counter. The percentage of radioactivity bound to cells was calculated.

Athymic nude mice bearing LS-180 human colorectal cancer cells were used for studying *in vivo* immunoreactivity and biodistribution of radiolabelled MAbs. Average weight of tumours was 0.46 g at 2 weeks after inoculation of LS-180 ($1 \times 10^7$ cells) subcutaneously into a rear flank. The mice were injected intravenously with approximately 400 ng of both $^{99m}$Tc-labelled and $^{125}$I-labelled MAbs. At 3 and 18 h post injection, the mice were anaesthetised and sacrificed, and the weight and radioactivity of major organs were measured. Biodistribution was presented as a percentage of the injected dose per gram of organ corrected for 20 g of body weight. Four to five mice with tumours of a defined size were examined in each group. Statistical analysis was made by Student's *t*-test. Scintigrams of tumour xenograft were obtained at 18 h after injection of high-dose $^{99m}$Tc-mNCA Ab or $^{99m}$Tc-chNCA Ab into mice with large tumours.

### *Binding to human granulocytes*

Venous blood was collected from a normal volunteer in a 50 ml syringe containing 0.4 ml of heparin and 7 ml of hydroxy ethylstarch, and sedimented at 1 × *g* for 60 min. Supernatant was collected and centrifuged at 1000 r.p.m. for 5 min to separate leucocytes. Leucocytes were resuspended in autologous plasma. A 10-fold dilution was repeated three times and the number of granulocytes were measured by flow cytometry and cytochemical analysis using Technicon (Technicon Instruments, Tarrytown, NY, USA). Radiolabelled MAbs (30 ng in 0.05 M PBS) were incubated with increasing numbers of granulocytes ($7.4 \times 10^3 - 7.4 \times 10^6$) in microcentrifuge tubes for 1 h at 25°C. The percentage of radioactivity bound to granulocytes was calculated in the same manner with the cell binding assay.

### *Clinical study*

Four patients (63 to 74 years of age) with metastatic bone tumour from prostate cancer were studied. All patients gave informed consents to participate in the study which was approved by the ethical committee of our university. $^{99m}$Tc-chNCA Ab (1110 MBq, 1 mg) was mixed with 100 ml physiological saline followed by the intravenous injection for 10 min. Temperature, blood pressure, heart rate, respiratory rate and subjective symptoms were monitored before, during and until 30 min after the infusion.

Scintigraphy was performed using a gamma camera (ZLC 7500, Siemens, IL, USA) fitted with a low-energy all-purpose collimator. On-line computer Scintipac 700 (Shimadzu, Kyoto, Japan) was used for processing the image data. Following dynamic imaging of abdomen anteriorly for 30 min from the beginning of injection, anterior and posterior planar images of the chest, abdomen and pelvis were obtained at 1, 4 and 24 h after injection.

### *Pharmacokinetics and immune response*

Blood samples were collected sequentially at 5, 10, 15, 20, 25, 30 and 60 min, 4 and 24 h after injection of $^{99m}$Tc-chNCA Ab. Radioactivity of whole-blood samples and plasma samples were counted with a gamma counter to determine the blood clearance of $^{99m}$Tc-chNCA Ab. High-pressure liquid chromatography (HPLC) analysis was performed using 5 min and 30 min plasma samples with a G3000SW column (Tosoh). Blood samples were also obtained at intervals from 2 to at least 19 weeks to investigate the serological immune response to the injected chNCA Ab.

Detection of HAMA in the serum samples after injection of $^{99m}$Tc-chNCA Ab was performed by the radioimmunoassay as reported previously (LoBuglio *et al.*, 1986; Khazaeli *et al.*, 1991). Serum samples (100 μl) were incubated with mNCA Ab-coated beads for 90 min at 25°C. After washing twice with purified water, $^{125}$I-mNCA Ab was added and incubated for 60 min at 25°C. Purified water was then added and unbound radioactivity was washed away. Radioactivity bound to beads was counted with a gamma counter. HAMA was determined as positive when bound radioactivity to beads was over the mean values plus three standard deviations of 20 normal individuals. For the assay of HACA, the same procedure was applied using chNCA Ab instead of mNCA Ab. The same criteria were applied to categorise as a positive HACA response. Sera obtained from 20 healthy individuals and five patients receiving $^{99m}$Tc-BW431/26 (Oriuchi *et al.*, 1995) were also examined, and their HAMA and HACA titres were determined. Three of five patients receiving $^{99m}$Tc-BW431/26 developed HAMA detectable with this method.

### *HPLC analysis of $^{99m}$Tc-chNCA Ab incubated with human serum*

In order to assess the interaction of $^{99m}$Tc-chNCA Ab with the human serum, $^{99m}$Tc-chNCA Ab was incubated with human serum *in vitro* for 1 h at 37°C and HPLC analysis was performed with a G3000SW column (Tosoh) eluted at a flow rate of 1 ml min$^{-1}$ and 0.5 ml fractionation. Doses of $^{99m}$Tc-chNCA Ab used for the incubation were: (1) 5 μg of $^{99m}$Tc-chNCA Ab with 20 μl of human serum, which was performed as one of the preclinical tests before the clinical trial; and (2) 5 ng of $^{99m}$Tc-chNCA Ab with 20 μl of human serum, which was performed to confirm the presence of high molecular weight complex after the clinical trial. The latter dosage (2) was calculated as equivalent to that of the clinical situation.

### Results

The cellulose acetate electrophoresis of $^{99m}$Tc-chNCA Ab showed that labelling efficiency was more than 96% and there was no evidence of colloid formation. Specific activities of $^{99m}$Tc-labelled mNCA Ab and chNCA Ab were calculated as 1.5 GBq mg$^{-1}$. The cell binding assay showed that the percentage binding of $^{99m}$Tc-chNCA Ab to LS-180 cells increased as the cell number increased (Figure 1, top) and it was almost comparable with $^{125}$I-chNCA Ab and $^{99m}$Tc-mNCA Ab. Percentage binding of $^{99m}$Tc- and $^{125}$I-labelled chNCA Ab to human granulocytes also increased in proportion to granulocyte numbers added, and 73.4% and 66.7% of bound radioactivity were obtained at granulocyte numbers of $7.4 \times 10^6$ per tube (Figure 1, bottom). $^{99m}$Tc-labelled chNCA Ab and mNCA Ab showed a slightly different biodistribution in athymic mice bearing LS-180 as shown in Table I. Clearance of $^{99m}$Tc-chNCA Ab from the circulation was faster than that of $^{99m}$Tc-mNCA Ab resulting in a lower radioactivity of $^{99m}$Tc-chNCA Ab (% injected dose per gram tissue) in all organs except kidney. Tumour–normal organ ratio was high with $^{99m}$Tc-chNCA Ab except tumour–kidney ratio. At 18 h after injection of $^{99m}$Tc-chNCA Ab, tumour–blood ratio was 1.74; tumour–liver, 2.73; tumour–muscle, 13.7; whereas the ratios for $^{99m}$Tc-chNCA Ab were 0.74, 1.62 and 6.89 respectively. Tumour–kidney ratios for $^{99m}$Tc-labelled chNCA Ab and mNCA Ab were 0.33 and 0.87 respectively. Scintigrams of athymic mice confirmed the results of biodistribution studies, showing a higher radioactivity in the tumour and kidney of mice administered with $^{99m}$Tc-chNCA Ab (Figure 2).

Four patients with bone metastases received $^{99m}$Tc-chNCA Ab and no adverse reaction was noted after the administration in these patients. Dynamic images obtained immediately after injection demonstrated intense radioactivity in the liver

**Figure 1** Immunoreactivity of $^{99m}$Tc-chNCA Ab (–●–), $^{125}$I-chNCA Ab (- -○- -), $^{99m}$Tc-mNCA Ab (- - -▲- - -), $^{125}$I-mNCA Ab (–△–), $^{99m}$Tc-labelled control murine Ab (–■–) and $^{125}$I-labelled control murine Ab (–□–) evaluated by *in vitro* binding to human colorectal cancer cells (top) and to human granulocytes (bottom).

**Figure 2** Scintigrams of athymic mice carrying human colorectal cancer cell xenograft (arrow) at 18 h after administration of $^{99m}$Tc-mNCA Ab (left) and $^{99m}$Tc-chNCA Ab (right). Arrowheads: kidneys.

**Table I** Biodistribution of $^{99m}$Tc- and $^{125}$I-labelled chNCA Ab and mNCA Ab in athymic mice carrying human colorectal cancer cells at 3 and 18 h after intravenous administration.

| | 3 h | | | | 18 h | | | |
|---|---|---|---|---|---|---|---|---|
| Organ | $^{99m}$Tc-chNCA (n = 4) | $^{125}$I-chNCA (n = 4) | $^{99m}$Tc-mNCA (n = 4) | $^{125}$I-mNCA (n = 4) | $^{99m}$Tc-chNCA (n = 5) | $^{125}$I-chNCA (n = 5) | $^{99m}$Tc-mNCA (n = 4) | $^{125}$I-mNCA (n = 4) |
| Blood | 16.7±2.0 | 17.7±0.9 | 20.8±1.4[a] | 17.5±0.7 | 2.6±0.5 | 5.4±1.1[b] | 6.7±0.8[a] | 5.5±0.9[a] |
| Liver | 4.8±0.5 | 6.0±1.2 | 9.1±1.2[b] | 6.6±1.1 | 2.3±1.0 | 1.8±0.4 | 6.5±0.9[a] | 2.4±0.7 |
| Kidney | 19.3±2.0 | 8.0±1.3[a] | 12.5±1.1[b] | 7.4±1.1[a] | 17.6±4.3 | 2.5±0.7[a] | 13.9±1.2 | 2.4±0.2[a] |
| Intestine | 3.2±0.5 | 2.3±0.7 | 2.3±0.2 | 2.5±0.5 | 2.1±0.6 | 1.4±0.9 | 2.1±0.4 | 1.1±0.3 |
| Stomach | 1.2±0.6 | 4.4±2.0 | 1.2±0.1 | 3.8±1.9 | 1.4±0.8 | 2.8±1.3 | 1.3±0.3 | 2.5±1.2 |
| Spleen | 3.9±1.3 | 6.2±3.0 | 7.2±1.4 | 6.4±1.4 | 1.5±0.4 | 2.1±0.5 | 3.5±1.6 | 2.1±0.8 |
| Lung | 7.5±3.2 | 8.7±4.1 | 9.0±5.1 | 8.2±4.3 | 1.8±0.8 | 3.0±1.0 | 2.6±0.5 | 2.2±0.5 |
| Muscle | 0.7±0.2 | 1.0±0.3 | 1.1±0.3 | 0.8±0.3 | 0.2±0.02 | 0.4±0.07[b] | 0.6±0.2[b] | 0.5±0.1[b] |
| Bone | 1.8±0.2 | 2.6±0.8 | 3.0±0.6 | 2.8±0.7 | 0.6±0.1 | 0.9±0.2 | 1.5±0.4[b] | 1.0±0.2[b] |
| Tumour | 7.7±1.3 | 8.7±1.0 | 10.0±0.8 | 7.6±0.9 | 5.4±2.7 | 6.8±3.8 | 9.8±1.3 | 6.7±0.8 |

[a]$P<0.001$ compared with $^{99m}$Tc-chNCA. [b]$P<0.01$ compared with $^{99m}$Tc-chNCA.
Data are shown as % injected dose per g of organs (mean ± s.e.).

(data not shown). Static images of a patient demonstrated remarkable accumulation of the tracer in liver accompanied by moderate accumulation in spleen, lung and kidney (Figure 3). Metastatic bone lesion could be seen as a photopenic area on 1 and 4 h posterior images (Figure 4). Blood clearance of the radioactivity in four patients demonstrated that the biphasic curve was found to be best fitted with the first-phase half-life of 6.4±1.1 min (mean±s.d.) and the second-phase half-life of 70.8±41.6 h (Figure 5). Figure 6 represents the HPLC elution profile of plasma samples of a patient obtained at 5 and 30 min after injection of $^{99m}$Tc-chNCA Ab. More than 60% of the plasma radioactivity was found at the first peak at around 29 min of the retention time corresponding to a high molecular weight material in the circulation.

HPLC analysis showed that there was the first peak before the second peak of $^{99m}$Tc-chNCA Ab under incubation condition similar to those pertaining to the clinical situation (5 ng of $^{99m}$Tc-chNCA Ab with 20 μl serum), which indicated the evidence of a high molecular weight complex formation. However, there was apparently no complex formation when $1 \times 10^3$-fold excess of $^{99m}$Tc-chNCA Ab was incubated with serum (5 μg of $^{99m}$Tc-chNCA Ab with 20 μl serum) as shown in Figure 7.

No patient showed positive HACA or HAMA response in their sera within 19 weeks after injection of $^{99m}$Tc-chNCA Ab, whereas 60% of patients produced HAMA in their sera after injection of the same dose of $^{99m}$Tc-BW431 26 (Figure 8).

**Figure 3** Anterior static images obtained 1, 4 and 24 h after administration of $^{99m}$Tc-chNCA Ab in a 64-year-old patient with metastatic bone tumour from prostate cancer. Intense radioactivity was seen in the liver.

**Figure 4** Posterior static images obtained 1 and 4 h after administration of $^{99m}$Tc-chNCA Ab and $^{99m}$Tc-HMDP bone scintigraphy in the same patient as Figure 3. Metastatic bone lesion can be seen as a vague photopenic area on $^{99m}$Tc-chNCA Ab images (arrows), and a hotspot on bone scintigraphy (arrowhead).

**Figure 5** Blood clearance of $^{99m}$Tc-chNCA Ab in four patients with metastatic bone tumour. A biphasic curve fitting was demonstrated with mean first-phase half-life of 6.4 min and second-phase half life of 70.8 h.

**Figure 6** HPLC elution profile of plasma drawn at 5 min (–▲–) and 30 min (- - -▲- - -) after the administration of $^{99m}$Tc-chNCA Ab.

**Figure 7** HPLC elution profiles of $^{99m}$Tc-chNCA Ab after incubation with normal human serum for 60 min. $^{99m}$Tc-chNCA Ab and normal human serum were incubated under two different chNCA Ab to serum ratios: 5 μg of $^{99m}$Tc-chNCA Ab with 20 μl serum (-●-); 5 ng of $^{99m}$Tc-chNCA Ab with 20 μl serum (- -○- -).

**Figure 8** Human anti-mouse antibody (HAMA) and human anti-chimera antibody (HACA) levels in serum of normal controls, patients receiving $^{99m}$Tc-chNCA Ab and $^{99m}$Tc-BW431/26, murine anti-CEA Ab. Dotted lines indicate the normal ranges obtained from healthy individuals.

### Discussion

Chimeric anti-NCA Ab was successfully labelled with $^{99m}$Tc with high specific activity of 1.5 GBq mg$^{-1}$. $^{131}$I and $^{111}$In have been conjugated with MAb and used for radioimmunodetection. $^{99m}$Tc is relatively new for conjugating MAb, especially for chimeric MAb. However, $^{99m}$Tc has several advantages for imaging. $^{99m}$Tc is easily available since it is produced by a generator system and photon energy is suitable for gamma camera imaging. The relatively short half-life of $^{99m}$Tc enables sufficient amount of injection dose to provide tomography with a high resolution and low statistical error.

Animal experiments showed that $^{99m}$Tc-chNCA Ab was accumulated in the xenografted tumour which expressed CEA. In contrast to animal studies, clinical examination revealed that most radioactivity was present in the liver immediately after intravenous injection of $^{99m}$Tc-chNCA Ab. Blood clearance of $^{99m}$Tc-chNCA Ab showed a biphasic clearance with the first-phase half-life of $6.4 \pm 1.1$ min, although reported mean half-lives of chimeric MAbs in humans were approximately 18 h (first phase) and 100 h (second phase), six times longer than those of murine MAbs (LoBuglio *et al.*, 1989; Meredith *et al.*, 1991). HPLC analysis of plasma from the patient who received $^{99m}$Tc-chNCA Ab revealed the formation of a high molecular weight complex in the circulation and there was no increment in free $^{99m}$Tc pertechnetate in the plasma detected by either whole body imaging or HPLC analysis. NCA has been found in various human tissues such as lung, spleen, granulocytes and also in the serum with a variety of molecular weights ranging from 50 to 160 kDa (von Kleist *et al.*, 1972; Bosslet *et al.*, 1985). *In vivo* instability of reduction-mediated $^{99m}$Tc-labelled Ab has been reported (Sakahara *et al.*, 1993) but no evidence of aggregation or complex formation was noted when $^{99m}$Tc-chNCA Ab was incubated with PBS (data not shown).

HPLC analysis of the diluted $^{99m}$Tc-chNCA Ab incubated with normal human serum *in vitro* demonstrated the same radioactivity peak corresponding to a high molecular weight complex (Figure 7). Serum samples, which were absorbed by anti-NCA Ab and then incubated with $^{99m}$Tc-chNCA Ab, showed only one peak which corresponded to $^{99m}$Tc-chNCA Ab (data not shown). From these results, increased radioactivity in the liver is supposed to be caused by the complex formation of injected $^{99m}$Tc-chNCA Ab with circulating NCA resulting in subsequent clearance by the reticuloendothelial system. Of note was the fact that a high molecular weight complex was only detected by HPLC under a certain incubation ratio of radiolabelled MAb to human serum. According to the recommendation of the Food and Drug Administration in USA (Center for Biologics Evaluation and Research, FDA 1993), $^{99m}$Tc-chNCA Ab was examined for binding to serum protein using HPLC by incubating $^{99m}$Tc-chNCA Ab with human serum as a preclinical safety testing, though the high molecular weight complex was hardly demonstrable probably because of the saturation of antigen-binding capacity present in the serum. On the contrary, when $^{99m}$Tc-chNCA Ab and human serum were incubated under the strict dilution ratio ($1 \times 10^3$-fold), which was the equivalent ratio of $^{99m}$Tc-chNCA Ab to serum in the circulation, then a high molecular weight complex was clearly demonstrated by HPLC.

The biodistribution profile observed in this study was different from other studies. The disparity between the results of the biodistribution of $^{131}$I-labelled MAb in man and mice has been reported (Ledermann *et al.*, 1993). In the report, most of the radioactivity was located in the bone marrow and spleen, since the radiolabelled MAb was bound to circulating granulocytes. Lack of immunodetection and complications associated with MAb cross-reactive with circulating cells had been reported (Dillman *et al.*, 1984).

Several authors have suggested that the presence of antigen in the blood does not significantly affect the results of imaging, though it would form a circulating immune complex (Goldenberg *et al.*, 1978; Mach *et al.*, 1980; Primus *et al.*, 1980). Pharmacokinetics and biodistribution of $^{111}$Inlabelled MAb which formed a circulating immune complex have been reported (Hnatowich *et al.*, 1987; Davidson *et al.*, 1991). In the present study, increased liver uptake of radiolabelled MAb was noted as in the previous studies, although the pharmacokinetics were different and imaging was not successful. Blood clearance of $^{99m}$Tc-chNCA Ab was much faster than in other studies and the mechanism of fast clearance and the liver accumulation of $^{99m}$Tc-chNCA Ab remains to be clarified.

None of the patients produced HAMA or HACA within 19 weeks after $^{99m}$Tc-chNCA Ab injection, although three of five patients who received the same 1 mg of $^{99m}$Tc-labelled murine Ab produced HAMA in their serum. There have been a few reports describing the immunogenicity of chimeric Abs. Chimeric Abs designated 17-1A, L6 and NR-LU-13 had low immunogenicity, whereas $^{131}$I-labelled chimeric Ab, B72.3, had considerable immunogenicity (Khazaeli *et al.*, 1991; LoBuglio *et al.*, 1989; Meredith *et al.*, 1991). In the latter paper, seven of 12 patients with metastatic colon cancer had an antibody response after intravenous injection of 3.4 to 6.9 mg of chimeric B72.3, and a small portion of the antibody response was directed to epitopes requiring the presence of both murine V-region and human CH-1/κ constant regions. These results may indicate that the immunogenicity of chimeric Abs may depend on the amino acid sequences of murine V-region.

In conclusion, chNCA Ab was stably labelled with $^{99m}$Tc pertechnetate and immunoreactivity was completely reserved with high-binding capacity to human granulocytes and CEA-expressing colon cancer cells. $^{99m}$Tc-chNCA Ab was safely administered to patients without generating HAMA or HACA response. In contrast to animal studies, however, circulating antigenic molecules reactive with chNCA Ab formed a high molecular weight complex immediately after the administration of $^{99m}$Tc-chNCA Ab and were taken up in the liver. For safety HPLC analysis should be performed before clinical radioimmunodetection or radioimmunotherapy by incubating radiolabelled MAb with human serum under strict conditions.

**Acknowledgements**

This study was supported in part by grants from the Ministry of Health and Welfare, and the Ministry of Education, Science and Culture, Japan.

### References

BAUM RP, HERTEL A, LORENTZ M, SCHWARZ A, ENCHE A AND HOR G. (1989). $^{99m}$Tc-labeled anti-CEA monoclonal antibody for tumour immunoscintigraphy: first clinical results. *Nucl. Med. Commun.*, **10**, 345–352.

BOSSLET K, LUBEN G, SCHWARZ A, HUNDT E, HARTHUS HP, SEILER FR, MUHRER C, KLOPPEL G, KAYSER K AND SEDLACEK HH. (1985). Immunohistochemical localization and molecular characteristics of three monoclonal antibody-defined epitopes detectable on carcinoembryonic antigen (CEA). *Int. J. Cancer*, **36**, 75–84.

BOULIANNE GL, HOZUMI N AND SHULMAN MJ. (1984). Production of chimeric mouse/human antibody. *Nature*, **312**, 643–646.

CENTER FOR BIOLOGICS EVALUATION AND RESEARCH, FDA. (1993). Points to consider in the manufacture and testing of monoclonal antibody products for human use. FDA: Rockville.

COURTENAY-LUCK N, EPENETOS AA, HALMAN KE, HOOKER G, HUGHES JMB, KRAUSZ T, LAMBERT J, LAVENDER JP, MACGREGOR WG, MONRO A, MYERS MJ, ORR JS, PEARSE EE, SNOOK D, WEBB B, BURCHELL J, DURBIN H, KEMSHEAD J AND TAYLOR-PAPADIMITRION J. (1984). Antibody guided irradiation of malignant lesions: three cases illustrating a new method of treatment. *Lancet*, **1**, 1441–1443.

DAVIDSON BR, BABICH J, YOUNG H, WADDINGTON W, CLARKE G, SHORT M, BOULOS P, STYLES J AND DEAN C. (1991). The effect of circulating antigen and radiolabelled stability on the biodistribution of an indium labelled antibody. *Br. J. Cancer*, **64**, 850–856.

DILLMAN RO, BEAUREGARD JC, SOBOL RE, ROYSTON I, BAERTHOLOMEW RM, HAGAN PH AND HALPERN SE. (1984). Lack of radioimmunodetection and complications associated with monoclonal anticarcinoembryonic antigen antibody cross-reactivity with an antigen on circulating cells. *Cancer Res.*, **44**, 2213–2218.

GOLDENBERG DM, DELAND F, KIM E, BENNETT S, PRIMUS FJ, VANNAGELL TR, ESTES N, DESIMONE P AND RAYBURN P. (1978). Use of radiolabelled antibodies to carcinoembryonic antigen for the detection and localisation of diverse cancer by external photoscanning. *N. Eng. J. Med.*, **298**, 1384–1388.

HNATOWICH DJ, GIONET M, RUSCKOWSKI M, SIEBECKER DA, ROCHE J, SHEALY D, HATTIS JA, WILSON J, MCGANN J, HUNTER RE, GRIFFIN T AND DOHERTY PW. (1987). Pharmacokinetics of $^{111}$In-labeled OC-125 antibody in cancer patients compared with 19-9 antibody. *Cancer Res.*, **47**, 6111–6117.

HUNTER WM AND GREENWOOD FC. (1962). Preparation of iodine-131-labelled growth hormone of high specific radioactivity. *Nature*, **194**, 495–496.

KHAZAELI MB, SALEH MN, LIU TP, MEREDITH RF, WHEELER RH, BAKER TS, KING D, SECHER D, ALLEN L, ROGERS K, COLCHER D, SCHLOM J, SHOCHAT D AND LOBUGLIO AF. (1991). Pharmacokinetics and immune response of $^{131}$I-chimeric mouse/human B72.3 (human $IgG_4$) monoclonal antibody in humans. *Cancer Res.*, **51**, 5461–5466.

KOGA H, KANDA H, NAKASHIMA M, WATANABE Y, ENDO K AND WATANABE T. (1990). Mouse–human chimeric monoclonal antibody to carcinoembryonic antigen (CEA): *in vitro* and *in vivo* activities. *Hybridoma*, **9**, 43–56.

LEDERMANN JA, MARSTON NJ, STAHEL RA, WAIBEL R, BUSCOMBE JR AND ELL PJ. (1993). Biodistribution and tumour localisation of $^{131}$I SWA11 recognising the cluster w4 antigen in patients with small cell lung cancer. *Br. J. Cancer*, **68**, 119–121.

LOBUGLIO AF, WHEELER RH, TRANG J, HAYNES A, ROGERS K, HARVEY EB, SUN L, GHRAYEB J AND KHAZAELI MB. (1989). Mouse/human chimeric monoclonal antibody in man: kinetics and immune response. *Proc. Natl Acad. Sci. USA*, **86**, 4220–4224.

LOCHER JT, SEYBOLD K, ANDRES RJ, SCHUBINGER PA, MACH JP AND BUCHEGGER F. (1986). Imaging of inflammatory and infectious lesions after injection of radiolabeled monoclonal antigranulocyte antibodies. *Nucl. Med. Commun.*, **7**, 659–670.

MACH JP, CARREL S, FORNI M, RITSCHARD J, DONATH A AND ALBERTO P. (1980). Tumor localization of radiolabeled antibodies against carcinoembryonic antigen in patients with carcinoma: a critical evaluation. *N. Engl. J. Med.*, **303**, 5–10.

MATHER SJ AND ELLISON D. (1990). Reduction-mediated technetium-99m labeling of monoclonal antibodies. *J. Nucl. Med.*, **31**, 692–697.

MEREDITH RF, LOBUGLIO AF, PLOTT WE, ORR RA, BREZOVICH IA, RUSSEL CD, HARVEY EB, YESTER MV, WAGNER AJ, SPENCER SA, WHEELER RH, SALEH MN, ROGERS KJ, POLANSKI A, SALTER MM AND KHAZAELI MB. (1991). Pharmacokinetics, immune response, and biodistribution of iodine-131-labeled chimeric mouse/human $IgG_1$, κ 17-1A monoclonal antibody. *J. Nuc. Med.*, **32**, 1162–1168.

MUNZ DL, SANDROCK D AND RILINGER N. (1990). Comparison of immunoscintigraphy and colloid scintigraphy of bone marrow. *Lancet*, **336**, 258–259.

ORIUCHI N, ENDO K, WATANABE N, SUGIYAMA S, ASAO T, TAKENOSHITA S, NAGAMACHI Y AND BAUM RP. (1995). Semi-quantitative SPECT tumor uptake of $^{99m}$Tc-labeled anti-CEA monoclonal antibody in colorectal tumor. *J. Nucl. Med.*, **36**, 679–683.

POTTER H, WEIR L AND LEDER P. (1984). Enhancer-dependent expression of human k immunoglobulin genes introduced into mouse pre-B lymphocytes by electroporation. *Proc. Natl Acad. Sci. USA*, **81**, 7161–7165.

PRIMUS FJ, BENNETT SJ, KIM EE, DELAND FH, ZAHN MC AND GOLDENBERG DM. (1980). Circulating immune complex in cancer patients receiving goat radiolocalizing antibodies to carcinoembryonic antigen. *Cancer Res.*, **40**, 497–501.

REULAND P, WINKER KH, HEUCHERT T, RUCK P, MULLER-SCHAUENBERG W, WELLER S AND FEINE U. (1991). Detection of infection in prospective orthopedic patients with technetium-99m-labeled monoclonal antibodies against granulocytes. *J. Nucl. Med.*, **32**, 2209–2214.

SAKAHARA H, SAGA T, ENDO K, HATTORI N, HOSONO M, KOBAYASHI H, SHIRATO M, YAMAMURO T, TOYAMA S, ARANO Y, YOKOYAMA A AND KONISHI J. (1993). *In vivo* instability of reduction-mediated $^{99m}$Tc-labeled monoclonal antibody. *Nucl. Med. Biol.*, **20**, 617-623.

SCHROFF RW, FOON KA, BEATTY SM, OLDHAM RK AND MORGAN AC JR. (1985). Human anti-murine immunoglobulin responses in patients receiving monoclonal antibody therapy. *Cancer Res.*, **45**, 879-885.

SHAWLER DL, BARTHOLOMEW RM, SMITH LM AND DILLMAN RO. (1985). Human immune response to multiple injections of murine monoclonal IgG. *J. Immunol.*, **135**, 1530-1535.

VON KLEIST S, CHAVANE LG AND BURTIN P. (1972). Identification of an antigen from normal human tissue that crossreacts with the carcinoembryonic antigen. *Proc. Natl Acad. Sci. USA*, **69**, 2492-2494.

WATANABE N, ORIUCHI N, SUGIYAMA S, KUROKI M AND MATSUOKA Y. (1994). Radioimmunoscintigraphy of colorectal cancer with Tc-99m-labeled murine anti-carcinoembryonic antigen antibody in athymic nude mice. *Ann. Nucl. Med.*, **8**, 23-30.